# The Mediating Effects of Job Satisfaction on the Association between Doctor-patient Relationship and OCB among Physicians in China

**Published:** 2018-05

**Authors:** Rui HAN, Lin WEI, Jingping LI, Ding ZHANG, Hongliang LI

**Affiliations:** 1. Institute of Ideological & Political Education, School of Marxism, Xi’an Jiaotong University, Xi’an, China; 2. Dept. of Political Science, Xi’an Jiaotong University, Xi’an, China; 3. Dept. of Hematology, Shaanxi People’s Hospital, Xi’an, China; 4. Second Affiliated Hospital, Xi’an Jiaotong University, Xi’an, China

**Keywords:** Job satisfaction, Doctor-patient relationship, OCB

## Abstract

**Background:**

The aim of this study was to investigate OCB among physicians in China and explore whether their job satisfaction mediates the association between doctor-patient relationship (DPR) and organizational citizenship behavior (OCB).

**Methods:**

This cross-sectional, questionnaire-based survey was conducted among 1400 physicians in Shaanxi, China in 2014. The subjects were selected using a multi-stage cluster sampling methodology. The self-administered questionnaires included OCB Scale, DDPRQ, and PJSQ. Hierarchical linear regression analysis was used to estimate the effects of job satisfaction on the association between DPR and OCB.

**Results:**

DPR negatively predicted four dimensions of OCB, including conscientiousness, sportsmanship, civic virtue, and altruism. DPR was negatively related to five job satisfaction dimensions, namely work satisfaction (WS), promotion satisfaction (PS), reward satisfaction (RS), supervision satisfaction (SS), and environment satisfaction (ES). WS was positively correlated with conscientiousness and civic virtue; PS and SS were positively related to all four OCB dimensions; RS was positively related with civic virtue and altruism, and ES was positively correlated with conscientiousness and civic virtue. WS and PS partially mediated the association between DPR and conscientiousness; PS and SS partially mediated the relation between DPR and sportsmanship; PS, SS, and ES mediated the association between DPR and civic virtue; and PS, RS and SS partially mediated the relation between DPR and altruism.

**Conclusion:**

Job satisfaction mediated the association between DPR and OCB among Chinese physicians. The poor DPR possibly reduce physicians’ job satisfaction, thereby causing a decline of OCB in hospitals. Therefore, DPR improvement and job satisfaction have a great potential to promote physicians’ job performance in China.

## Introduction

In China, physicians practicing in hospitals are the major providers of medical services, playing a vital role in determining health care quality (1).

To improve the overall performance of healthcare delivery, it is important to promote the organizational citizenship behavior (OCB) of physicians. Doctor-patient relationship (DPR) is a robust predictor of physicians’ OCB (2, 3). Physician-patient communication directly influences the OCB of specialty physicians (4). Although DPR may influence OCB indirectly through intervening job satisfaction (5, 6), the triadic relationship among DPR, OCB, and job satisfaction remains largely unknown and lacks empirical evidence (7).

OCB is considered discretionary work behavior that improves the effective functioning of an organization (8). OCB includes the following dimensions: conscientiousness, sportsmanship, civic virtue, altruism, and courtesy (9). There are increasing data supporting the notion that, in hospital setting, OCB is capable to improve the quality of care delivered, facilitate achievement of the hospital’s goals, and enhance physicians’ job performance, reflected by more efficient medical care and betterment of relations with patients and colleagues (6,10).

DPR is described as a therapeutic relationship in which physicians build an alliance with patients (11). Effective physician-patient interaction is related to a wide range of positive work behaviors, such as increased diagnostic accuracy (12), improved use of health resources and better medical care (13). DPR may have both direct and indirect effects on physicians’ OCB (4, 7).

Job satisfaction is identified as an evaluative state that expresses contentment with and positive feeling about one’s job (14). Physician job satisfaction may be affected by at least five factors, including job demands, job control, collegial support, income, and incentives (15). Doctor-patient interaction is a major source of satisfaction in physicians (16,17). And DPR has strong power in predicting physicians’ job satisfaction (18). In addition, job satisfaction has been found to be associated with OCB among hospital employees (19, 20). Increases in physician job satisfaction often lead to greater organizational performance for patient care (21).

The potential impact of job satisfaction on the association between DPR and OCB has not been examined in physicians. The aim of this study was to investigate OCB among physicians in China and determine whether the association between DPR and OCB is mediated by job satisfaction.

## Materials and Methods

### Participants

This cross-sectional study was conducted in Shaanxi Province (population, 37.75 million), China, between Aug and Sep 2014. Participants were selected using a multi-stage cluster-sampling method. At the first stage, one city in each region of the province was randomly selected. Then, two large general hospitals (>500 beds) were randomly selected if the sampling city was a capital city, or one large general hospital was randomly selected from each city. At the third stage, 50% of the physicians from each hospital were randomly sampled. In total, 1400 physicians were selected from six large general hospitals in five cities. Effective responses were obtained from 1017 individuals, which formed the study sample for analyses.

### Measurements

A) OCB questionnaire: The OCB scale used in this study was previously reported (22). It was a 24-item tool, measuring the five dimensions of OCB. This instrument used a five-point Likert scale with anchors of 1 to 5. Total score measured OCB, and higher scores indicated better OCB. In this study, the four factors that emerged after factor analysis were conscientiousness, sportsmanship, civic virtue, and altruism. The Cronbach’s alpha value of the OCB scale was 0.894. After revising three items, the results of confirmatory factor analysis (CFA) showed that the fit of the four-factor model was acceptable, goodness-of-fit index (GFI) = 0.953, normed fit index (NFI) = 0.940, comparative fit index (CFI) = 0.957, and root mean square error of approximation (RMSEA) = 0.049.

B) Difficult Doctor-Patient Relationship Questionnaire (DDPRQ): The DPR was measured with the 10-item version of the DDPRQ, which was proposed (23) to reflect physicians’ perception of difficulties in the DPR. Each item was scored from 1 to 6, except for items 1, 7, and 9, reverse-scored. Lower scores reflected less difficulty in caring for patients than higher scores. The Cronbach’s alpha value of the DDPRQ was 0.773, and CFA confirmed that the DDPRQ had a satisfactory goodness-of-fit (GFI = 0.985, NFI = 0.972, CFI = 0.981, RMSEA = 0.046).

C) Physicians’ Job Satisfaction Questionnaire (PJSQ): The PJSQ was used to measure job satisfaction (24). The total scale consisted of 58 items, and each item was rated from 1 to 5, except for items 5, 7, 51, 52, and 53, reverse-scored. Higher scores indicated greater job satisfaction. The Cronbach’s alpha value of the PJSQ was 0.965, and CFA confirmed that the PJSQ had a satisfactory goodness-of-fit (GFI = 0.943, NFI = 0.961, CFI = 0.975, RMSEA = 0.040).

### Ethical Consideration

This survey and protocols were reviewed and approved by the Ethics Committee, Xi’an Jiaotong University. Verbal informed consent was obtained from each participant after the study objective, sampling methods and analysis, and reporting procedure were explained to eligible physicians. Verbal informed consent was considered appropriate over written informed consent because this anonymous study was very low risk and among educated healthcare professionals.

### Statistical Analysis

The research variables were compared between various groups by gender, age, education, and length of employment by an independent-sample t-test and one-way analysis of variance. Pearson’s correlation analysis was used to examine correlations among research variables. Hierarchical linear regression analysis was applied to estimate the mediation effect of job satisfaction on the association between DPR and OCB. According to the criteria, to establish mediation, the following conditions must hold: First, the independent variable must affect the dependent variable; second, the independent variable must be shown to affect the mediator; third, the mediator must affect the dependent variable; fourth, the effect of the independent variable on the dependent variable must be reduced when the mediator is included (25).

The concerned variables were entered in the hierarchical linear regression equation in three steps. In the first step, demographic characteristics (gender, age, education, and length of employment) were added as the control variables. The significant DPR was included as the independent variable in the second step. All significant job satisfaction variables were involved as the mediators in the third step.

## Results

The participant characteristics and distributions of OCB dimensions for the categorical variables are indicated in Table 1. 51.43% of the participants were females; 49.16% were 26–35 yr old; 68.73% had a graduate or higher degree; and 39.63% had up to 5 yr of work experience.

**Table 1:** Participant characteristics and the distributions of OCB dimensions by categorical variable

***Category***	***Subcategory***	***N (%)***	***OCB Dimensions***			
			***Conscientiousness*** ***Mean (SD)***	***Sportsmanship*** ***Mean (SD)***	***Civic virtue*** ***Mean (SD)***	***Altruism*** ***Mean (SD)***
Gender	Male	494(48.57)	2.99(0.63)	3.01(0.54)	2.33(0.52)	2.79(0.37)
	Female	523(51.43)	3.09(0.63)	3.09(0.52)	2.27(0.50)	2.86(0.38)
*t*			−2.441	−2.342	1.737	−2.677
*P*			0.015	0.019	0.083	0.008
Age (yr)	≤25	123(12.09)	2.95(0.66)	2.93(0.55)	2.23(0.51)	2.80(0.40)
	26–35	500(49.16)	3.01(0.64)	3.02(0.53)	2.25(0.52)	2.83(0.40)
	36–45	260(25.57)	3.09(0.60)	3.13(0.55)	2.38(0.49)	2.84(0.34)
	46–55	116(11.41)	3.07(0.64)	3.12(0.44)	2.38(0.49)	2.85(0.38)
	>55	18(1.77)	3.39(0.54)	3.26(0.45)	2.63(0.41)	2.95(0.33)
*F*			2.603	4.700	6.372	0.857
*P*			0.035	0.001	0.000	0.489
Education	Junior college or lower	38(3.74)	3.13(0.70)	3.06(0.60)	2.24(0.43)	2.85(0.44)
	College	280(27.53)	3.10(0.68)	3.09(0.51)	2.32(0.51)	2.86(0.37)
	Graduate or higher	699(68.73)	3.01(0.61)	3.04(0.53)	2.30(0.51)	2.82(0.38)
*F*			2.554	0.703	0.407	1.286
*P*			0.078	0.495	0.666	0.277
length of employment (yr)	≤5	403(39.63)	2.97(0.64)	2.96(0.52)	2.23(0.52)	2.80(0.40)
	6–10	273(26.84)	3.09(0.61)	3.10(0.60)	2.30(0.54)	2.83(0.38)
	11–15	128(12.59)	3.04(0.67)	3.05(0.44)	2.40(0.49)	2.84(0.33)
	16–20	93(9.14)	3.01(0.58)	3.11(0.44)	2.34(0.45)	2.85(0.33)
	>20	120(11.80)	3.16(0.63)	3.20(0.47)	2.42(0.44)	2.90(0.39)
*F*			2.681	6.309	5.301	2.664
*P*			0.030	0.000	0.000	0.026

There were significant differences in all four OCB dimensions for the length of employment (all *P*<0.05). The conscientiousness (*P*<0.05), sportsmanship (*P*<0.01) and civic virtue (*P*<0.001) scores differed across age groups. However, no significant difference was observed among the age groups with respect to altruism. Between genders, there were significant differences in scores for conscientiousness (*P*<0.05), sportsmanship (*P*<0.05), and altruism (*P*<0.01), however, there was no significant difference in the score for civic virtue. There were no significant differences in all four OCB dimensions among different education groups.

The correlations between the research variables are shown in Table 2. DPR was negatively related to all five-job satisfaction dimensions and all four OCB dimensions, respectively. WS was positively correlated with conscientiousness (*r* =0.140, *P*<0.01) and civic virtue (*r* =0.264, *P*<0.01), respectively. PS and SS were positively related to all four OCB dimensions, respectively. RS was positively related with civic virtue (*r* =0.279, *P*<0.01) and altruism (*r*=0.101, *P*<0.01), respectively. ES was positively correlated with conscientiousness (*r* =0.084, *P*<0.01) and civic virtue (*r* =0.303, *P* < 0.01), respectively.

**Table 2:** Means, standard deviations (SD) and correlations of DPR, job satisfaction, and OCB variables

	***Mean (SD)***	***1***	***2***	***3***	***4***	***5***	***6***	***7***	***8***	***9***
DPR										
1. Doctor–patient relationship Job satisfaction	2.22(0.51)									
2. Work satisfaction	2.24(0.64)	−0.196**								
3. Promotion satisfaction	2.27(0.58)	−0.169**	0.509**							
4. Reward satisfaction	2.16(0.85)	−0.176**	0.620**	0.650**						
5. Supervision satisfaction	2.30(0.56)	−0.153**	0.584**	0.627**	0.606**					
6. Environment satisfaction OCB	2.20(0.90)	−0.175**	0.512**	0.503**	0.623**	0.587**				
7. Conscientiousness	3.04(0.63)	−0.144**	0.140**	0.158**	0.061	0.128**	0.084			
8. Sportsmanship	3.05(0.53)	−0.134**	0.032	0.078*	0.047	0.100**	0.051	0.376**		
9. Civic virtue	2.30(0.51)	−0.142**	0.264**	0.387**	0.279**	0.319**	0.303**	0.359**	0.273	
10. Altruism	2.83(0.38)	−0.203**	0.009	0.082**	0.101**	0.079*	0.052	0.438**	0.563	0.275**

* *P* <0.05,

** *P* <0.01 (two-tailed)

The results of hierarchical linear regression analysis are presented in Table 3.

**Table 3:** Results of Hierarchical linear regression analysis (n =1017; beta coefficients were presented)

	***Conscientiousness***			***Sportsmanship***			***Civic virtue***			***Altruism***		
	***Step 1***	***Step 2***	***Step 3***	***Step 1***	***Step 2***	***Step 3***	***Step 1***	***Step 2***	***Step 3***	***Step 1***	***Step 2***	***Step 3***
Gender	−0.074*	−0.076	−0.072	−0.081	−0.082	−0.079	0.044	0.043	0.042	−0.053	−0.056	−0.057
Age(yr)	0.115	0.112	0.102	0.060	0.058	0.064	0.105	0.102	0.055	0.022	0.026	0.018
Education	−0.067*	−0.069	−0.059	−0.018	−0.020	−0.017	−0.009	−0.011	0.028	−0.026	−0.029	−0.028
Length of employment	0.098**	0.078	0.073	0.090**	0.097	0.097	0.132***	0.136***	0.144***	0.099	0.108**	0.104**
Doctor-patient relationship		−0.148***	−0.120***		−0.139***	−0.116***		−0.145***	−0.063		−0.208***	−0.169***
Work satisfaction			0.118**			0.046			0.050			0.008
Promotion satisfaction			0.162***			0.125			0.282***			0.191***
Reward satisfaction			0.061			0.054			0.056			0.293***
Supervision satisfaction			0.043			0.231***			0.101			0.171***
Environment satisfaction			0.009			0.058			0.145***			0.057
*R*^2^	0.019	0.041	0.075	0.026	0.046	0.097	0.023	0.044	0.214	0.015	0.058	0.124
Δ*R*^2^	0.019	0.022	0.034	0.026	0.020	0.051	0.023	0.021	0.170	0.015	0.043	0.066
*F*	4.797**	8.550***	8.140***	6.884***	9.716***	10.802***	5.907***	9.286***	27.377***	3.753**	12.384***	14.238***

* *P* <0.05,

** *P* <0.01,

*** *P* <0.001 (two-tailed)

To test the mediation effect of job satisfaction on the association between DPR and OCB, regression analyses with three steps were performed. First, the control variables significantly predicted conscientiousness (*R*^2^ = 0.019), sportsmanship (*R*^2^ = 0.026), civic virtue (*R*^2^ = 0.023), and altruism (*R*^2^ = 0.015), respectively. Second, DPR significantly predicted conscientiousness (β = −0.148, *P*<0.001; Δ*R*^2^ = 0.022), sportsmanship (β = −0.139, *P*<0.001; Δ*R*^2^ = 0.020), civic virtue (β = −0.145, *P*<0.001; Δ*R*^2^ = 0.021), and altruism (β = −0.208, *P* < 0.001; Δ*R*^2^ = 0.043), respectively. Third, WS and PS positively predicted conscientiousness (WS: β = 0.118, *P* < 0.01; PS: β = 0.162, *P*<0.001; Δ*R*^2^ = 0.034); PS and SS positively predicted sportsmanship (PS: β = 0.125, *P*<0.01; SS: β = 0.231, *P*<0.001; Δ*R*^2^ = 0.051); PS, SS, and ES positively predicted civic virtue (PS: β = 0.282, *P*<0.001; SS: β = 0.101, *P*<0.05; ES: β = 0.145, *P* < 0.001; Δ*R*^2^ = 0.170); PS, RS, and SS positively predicted altruism (PS: β = 0.191, *P* < 0.001; RS: β = 0.293, *P* < 0.001; SS: β = 0.171, *P*<0.001; Δ*R*^2^ = 0.066). When calculated including WS and PS, the association between DPR and conscientiousness was significantly diminished (β: from −0.148 to −0.120, *P*<0.001); in case PS and SS were included, the relationship between DPR and sportsmanship was significantly attenuated (β: from −0.139 to −0.116, *P*<0.001); when PS, SS, and ES were included, the association between DPR and civic virtue was significantly removed (β: from −0.145 to −0.063, *P*<0.001); if calculated including PS, RS, and SS, the relationship between DPR and altruism was significantly weakened (β: from −0.208 to −0.169, *P*<0.001). In addition, DPR was significantly correlated to WS, PS, RS, SS and ES (*P*<0.01). Therefore, WS and PS partially mediated the association between DPR and conscientiousness; PS and SS played partially mediating roles in the relation between DPR and sportsmanship; PS, SS, and ES mediated the association between DPR and civic virtue; PS, RS, and SS partially mediated the relation between DPR and altruism.

## Discussion

In this study, most Chinese physicians frequently demonstrated four kinds of OCB, including conscientiousness, sportsmanship, civic virtue, and altruism. This result was similar to study among hospital nurses (26). Health policy makers and administrators should realize the significance of OCBs, and make effects to attract and retain physicians exhibiting these behaviors.

According to the results of this study, the relationship between DPR and the five dimensions of Chinese physicians’ job satisfaction was statistically significant. These findings were in accordance with previous studies (27, 28). A better DPR was correlated with higher job satisfaction among healthcare staff (29). In recent years, DPR in Chinese healthcare industry is increasingly worsened. Insults and violent attacks have become common strategies of disappointed patients to fight against doctors (30). This problem can be explained in part by the geographical inequity of health resources, super-specialization of medicine, and high medical fees for ordinary people (31, 32). Furthermore, from the perspective of the physicians, lack of communication skills, usage of difficult medical terms, and failure in thoroughly comprehending patients’ complaints also hinder a good DPR (33). Eventually, in China, the ordinary patients have limited access to a reliable and stable healthcare service in public hospitals. The short-term relationship between under-training physicians (interns and residents) and the patients may be further disrupted by frequent rotations and shift changes, resulting in patients’ disappointment.

The association between DPR and OCB among Chinese physicians was demonstrated in this study. The negative effects of difficult DPR on conscientiousness, sportsmanship, civic virtue, and altruism were statistically significant. These results were parallel to a previous study, which revealed that interpersonal communication had significant correlation with OCB and its four dimensions in hospitals (34). Hence, effective interventions to enhance physicians’ work performance should be established based on a good DPR. Several aspects on the doctor’s part should be emphasized. These aspects include understanding patients’ needs and expectations better, showing respect, empathy, and interest in patients’ ideas, fears, and opinions, accepting patients’ wish to share decisions, and passing on clear and adequate information to patients (35).

We clarified the positive relationship between the five dimensions of job satisfaction and four kinds of OCB behaviors. These findings were consistent with the previous studies, which showed significant positive association between specific facets of job satisfaction and different dimensions of OCB in American and Thai medical staff (19, 20). Among Chinese physicians, high job satisfaction seems to make them exhibit more OCBs, which potentially promote the organizational efficiency of the hospital. Thus, hospital management should focus on elevating physicians’ job satisfaction by improving their working condition, promotion, pay, and supervision.

This study was the first to demonstrate that job satisfaction mediates the association between DPR and OCB among Chinese physicians. Apart from the direct effect of DPR on OCB behaviors, DPR also affects OCB through the mediation of job satisfaction. Two primary reasons should be involved to comprehend this triadic relationship. On the one hand, due to the norm of reciprocity, people tend to reciprocate toward others who help or benefit them (36). If patients provide good compliance, trust, and support where physicians experience job satisfaction, the physicians may reciprocate the favor. On the other hand, ample psychological studies have illustrated that persons who experience higher levels of job satisfaction tend to engage in citizenship behaviors (37, 38). Therefore, hospital administrators should develop strategies to increase physicians’ job satisfaction in the workplace. Additionally, hospital managers develop a healthy climate in which physicians and patients trust and respect each other mutually.

## Conclusion

DPR was significantly associated with four dimensions of OCB and five job satisfaction dimensions, respectively. WS, along with PS, partially mediated the association between DPR and conscientiousness. PS and SS played partially mediating roles in the relationship between DPR and sportsmanship. PS, SS, and ES were mediators between DPR and civic virtue. In addition, PS, RS, and SS partially mediated the relation between DPR and altruism. Altogether, the difficult DPR possibly reduce physicians’ job satisfaction, thereby causing a decline of OCB in hospitals. Therefore, DPR improvement and job satisfaction have a great potential to promote physicians’ job performance in China.

## Ethical considerations

Ethical issues (Including plagiarism, informed consent, misconduct, data fabrication and/or falsification, double publication and/or submission, redundancy, etc.) have been completely observed by the authors.

## References

[1] Huang MC Liu TC (2010). The impact of external environment and self-serving motivation on physician’s organizational citizenship behaviors. Journal of Behavioral Studies in Business, 2: 1–10.

[2] Ong LM De Haes JC Hoos AM Lammes FB (1995). Doctor-patient communication: A review of the literature. Soc Sci Med, 40 (7): 903–18. 7792630 10.1016/0277-9536(94)00155-m

[3] Matusitz J Spear J (2014). Effective doctor–patient communication: An updated examination. Soc Work Public Health, 29 (3): 252–66. 24802220 10.1080/19371918.2013.776416

[4] Ruiz-Moral R Perez Rodriguez E Perula de Torres LA De la Torre J (2006). Physician-patient communication: A study on the observed behaviours of specialty physicians and the ways their patients perceive them. Patient Educ Couns, 64 (1–3): 242–8. 16597495 10.1016/j.pec.2006.02.010

[5] Lin CT Chang CS (2015). Job Satisfaction of Nurses and Its Moderating Effects on the Relationship Between Organizational Commitment and Organizational Citizenship Behaviors. Res Theory Nurs Pract, 29 (3): 226–44. 26502558 10.1891/1541-6577.29.3.226

[6] Chahal H Mehta S (2010). Antecedents and consequences of organisational citizenship behaviour (OCB): A conceptual framework in reference to health care sector. Journal of Services Research, 10 (2): 25–44.

[7] Chu CI Lee MS Hsu HM Chen IC (2005). Clarification of the antecedents of hospital nurse organizational citizenship behavior-An example from a Taiwan regional hospital. J Nurs Res, 13(4): 313–24. 16372242

[8] Organ DW Podsakoff PM MacKenzie SB (2005). Organizational citizenship behavior: Its nature, antecedents, and consequences: Sage Publications.

[9] Podsakoff NP Whiting SW Podsakoff PM Blume BD (2009). Individual- and organizational-level consequences of organizational citizenship behaviors: A meta-analysis. J Appl Psychol, 94 (1): 122–41. 19186900 10.1037/a0013079

[10] Bahrami MA Montazeralfaraj R Gazar SH Tafti AD (2014). Relationship between Organizational Perceived Justice and Organizational Citizenship Behavior among an Iranian Hospital’s Employees, 2013. Electron Physician, 6 (2): 838–44. 25763156 10.14661/2014.838-844 PMC4324267

[11] Heritage J Maynard DW (2006). Problems and prospects in the study of physician-patient interaction: 30 years of research. Annu Rev Sociol, 32: 351–74.

[12] Marvel MK Epstein RM Flowers K Beckman HB (1999). Soliciting the patient’s agenda: Have we improved? JAMA, 281 (3): 283–7. 9918487 10.1001/jama.281.3.283

[13] Alamo MM Moral RR Perula de Torres LA (2002). Evaluation of a patient-centred approach in generalized musculoskeletal chronic pain/fibromyalgia patients in primary care. Patient Educ Couns, 48 (1): 23–31. 12220747 10.1016/s0738-3991(02)00095-2

[14] Judge TA Kammeyer-Mueller JD (2012). Job attitudes. Annu Rev Psychol, 63: 341–67. 22129457 10.1146/annurev-psych-120710-100511

[15] Scheurer D McKean S Miller J Wetterneck T (2009). U.S. physician satisfaction: A systematic review. J Hosp Med, 4 (9): 560–8. 20013859 10.1002/jhm.496

[16] Wallace JE Lemaire J (2007). On physician well being-you’ll get by with a little help from your friends. Soc Sci Med, 64 (12): 2565–77. 17451854 10.1016/j.socscimed.2007.03.016

[17] Solomon J (2008). How strategies for managing patient visit time affect physician job satisfaction: A qualitative analysis. J Gen Intern Med, 23 (6): 775–80. 18365288 10.1007/s11606-008-0596-y PMC2517888

[18] Zhang YM Feng XS (2011). Empirical study on determinants of physicians’ job satisfaction in urban public medical institutions. Chin Health Resources, 14: 77–9.

[19] Bolon DS (1997). Organizational citizenship behavior among hospital employees: A multidimensional analysis involving job satisfaction and organizational commitment. Hosp Health Serv Adm, 42(2): 221–41. 10167456

[20] Intaraprasong B Dityen W Krugkrunjit P Subhadrabandhu T (2012). Job satisfaction and organizational citizenship behavior of personnel at one university hospital in Thailand. J Med Assoc Thai, 95(Suppl 6): S102–8. 23130495

[21] Williams ES Manwell LB Konrad TR Linzer M (2007). The relationship of organizational culture, stress, satisfaction, and burnout with physician-reported error and suboptimal patient care: Results from the MEMO study. Health Care Manage Rev, 32 (3): 203–12. 17666991 10.1097/01.HMR.0000281626.28363.59

[22] Podsakoff PM MacKenzie SB Moorman RH Fetter R (1990). Transformational leader behaviors and their effects on followers’ trust in leader, satisfaction, and organizational citizenship behaviors. The Leadership Quarterly, 1 (2): 107–42.

[23] Hahn S Kroenke K Spitzer R (1996). The difficult patient: Prevalence, psychopathology, and functional impairment (vol 11, pg 1, 1996). BLACKWELL SCIENCE INC 238 MAIN ST, CAMBRIDGE, MA 02142. pp. 191. 10.1007/BF02603477 8691281

[24] Zhang YM Feng XS (2011). Confirmatory factor analysis of job satisfaction structure of physicians from public medical institutions. Chinese Journal of Health Statistics, 28 (1): 29–32.

[25] Baron RM Kenny DA (1986). The moderator–mediator variable distinction in social psychological research: Conceptual, strategic, and statistical considerations. J Pers Soc Psychol, 51 (6): 1173–82. 3806354 10.1037//0022-3514.51.6.1173

[26] Dargahi H Alirezaie S Shaham G (2012). Organizational citizenship behavior among Iranian nurses. Iran J Public Health, 41 (5): 85–90. PMC3468989 23113181

[27] Mo XT Xu LZ Luo HW (2015). Medical professional perceived doctor-patient relationship, job satisfaction and turnover intention. Chin J Clin Psychol, 23: 141–6.

[28] Daghio MM Ciardullo AV Cadioli T (2003). GPs’ satisfaction with the doctor–patient encounter: Findings from a community-based survey. Fam Pract, 20(3): 283–8. 12738697 10.1093/fampra/cmg309

[29] Lu Y Hu XM Huang XL (2016). Job satisfaction and associated factors among healthcare staff: A cross-sectional study in Guangdong Province, China[J]. BMJ Open, 6(7): e011388. 10.1136/bmjopen-2016-011388 PMC4964254 27436667

[30] Wang XQ Wang XT Zheng JJ (2012). How to end violence against doctors in China. Lancet, 380 (9842): 647–8. 22901881 10.1016/S0140-6736(12)61367-1

[31] Hou X Xiao L (2012). An analysis of the changing doctor-patient relationship in China. J Int Bioethique, 23 (2): 83–94. 22924194 10.3917/jib.232.0083

[32] Banerjee A Sanyal D (2012). Dynamics of doctor-patient relationship: A cross-sectional study on concordance, trust, and patient enablement. J Family Community Med, 19 (1): 12–9. 22518353 10.4103/2230-8229.94006 PMC3326765

[33] Wu H Zhao X Fritzsche K (2015). Quality of doctor-patient relationship in patients with high somatic symptom severity in China. Complement Ther Med, 23 (1): 23–31. 25637149 10.1016/j.ctim.2014.12.006

[34] Keldbari SB Alipour HR (2011). Organizational citizenship behavior and employees social capital case study Rasht Hospitals. Australian Journal of Basic and Applied Sciences, 5 (8): 1185–93.

[35] Dormohammadi T Asghari F Rashidian A (2010). What do patients expect from their physicians?. Iran J Public Health, 39 (1): 70–77. PMC3468967 23112992

[36] Baker WE Bulkley N (2014). Paying it forward vs. rewarding reputation: Mechanisms of generalized reciprocity. Organization Science, 25 (5): 1493–1510.

[37] McNeely BL Meglino BM (1994). The role of dispositional and situational antecedents in prosocial organizational behavior: An examination of the intended beneficiaries of prosocial behavior. J Appl Psychol, 79 (6): 836–44.

[38] Ziegler R Schlett C Casel K Diehl M (2012). The role of job satisfaction, job ambivalence, and emotions at work in predicting organizational citizenship behavior. Journal of Personnel Psychology, 11 (4): 176–90.

